# Phytopharmacological Evaluation of Different Solvent Extract/Fractions From *Sphaeranthus indicus* L. Flowers: From Traditional Therapies to Bioactive Compounds

**DOI:** 10.3389/fphar.2021.708618

**Published:** 2021-10-27

**Authors:** Hafiz Ibtesam Ahmad, Muhammad Faisal Nadeem, Haji Muhammad Shoaib Khan, Muhammad Sarfraz, Hammad Saleem, Umair Khurshid, Marcello Locatelli, Muhammad Ashraf, Naveed Akhtar, Syafiq Asnawi Zainal Abidin, Adel Alghamdi

**Affiliations:** ^1^ Department of Pharmacy, The Islamia University of Bahawalpur, Bahawalpur, Pakistan; ^2^ Institute of Pharmaceutical Sciences (IPS), University of Veterinary & Animal Sciences (UVAS), Lahore, Pakistan; ^3^ College of Pharmacy, Al Ain University, Al Ain, United Arab Emirates; ^4^ Bahawalpur College of Pharmacy, Bahawalpur Medical and Dental College, Bahawalpur, Pakistan; ^5^ Department of Pharmacy, University G. d'Annunzio of Chieti-Pescara, Chieti, Italy; ^6^ Department of Chemistry, The Islamia University of Bahawalpur, Bahawalpur, Pakistan; ^7^ Jeffrey Cheah School of Medicine and Health Sciences, Liquid Chromatography Mass Spectrometry (LCMS) Platform, Monash University, Bandar Sunway, Malaysia; ^8^ Department of Pharmaceutical Chemistry, Faculty of Clinical Pharmacy, Albaha University, Albaha, Saudi Arabia

**Keywords:** *Sphaeranthus indicus*, phytochemicals, antioxidant, enzyme inhibition, UHPLC-MS, HPLC-PDA

## Abstract

*Sphaeranthus indicus* L. is a medicinal herb having widespread traditional uses for treating common ailments. The present research work aims to explore the in-depth phytochemical composition and *in vitro* reactivity of six different polarity solvents (methanol, *n*-hexane, benzene, chloroform, ethyl acetate, and *n*-butanol) extracts/fractions of *S. indicus* flowers. The phytochemical composition was accomplished by determining total bioactive contents, HPLC-PDA polyphenolic quantification, and UHPLC-MS secondary metabolomics. The reactivity of the phenolic compounds was tested through the following biochemical assays: antioxidant (DPPH, ABTS, FRAP, CUPRAC, phosphomolybdenum, and metal chelation) and enzyme inhibition (AChE, BChE, α-glucosidase, α-amylase, urease, and tyrosinase) assays were performed. The methanol extract showed the highest values for phenolic (94.07 mg GAE/g extract) and flavonoid (78.7 mg QE/g extract) contents and was also the most active for α-glucosidase inhibition as well as radical scavenging and reducing power potential. HPLC-PDA analysis quantified rutin, naringenin, chlorogenic acid, 3-hydroxybenzoic acid, gallic acid, and epicatechin in a significant amount. UHPLC-MS analysis of methanol and ethyl acetate extracts revealed the presence of well-known phytocompounds; most of these were phenolic, flavonoid, and glycoside derivatives. The ethyl acetate fraction exhibited the highest inhibition against tyrosinase and urease, while the *n*-hexane fraction was most active for α-amylase. Moreover, principal component analysis highlighted the positive correlation between bioactive compounds and the tested extracts. Overall, *S. indicus* flower extracts were found to contain important phytochemicals, hence could be further explored to discover novel bioactive compounds that could be a valid starting point for future pharmaceutical and nutraceuticals applications.

## 1 Introduction

Plants are essential for human life for survival, shelter, food, and medicine. Great interest has developed for an investigation into medicinal plants as a novel source of enzymes inhibitors, natural antioxidant compounds, and treating a number of common ailments (Phumthum et al., 2018). Since times immemorial, as recorded for Mesopotamia, plants have been a major source to alleviate illness and suffering. Much awareness has improved since then. Botanists have advocated the traditional use of plants as a whole or as ingredients, which have been mainly based on their safety, availability, and affordability (Abioye et al., 2019). Among the naturopathies, these are the reasons which have increased the interest for elucidation of biological potential as well as chemical constituents of plants (Mamadalieva et al., 2019).

The knowledge of organic chemistry and growth in the pharmaceutical field have led toward the discovery of synthetic medicine but have also impacted the side effects and the high cost as a negative image of these discovered drugs. This ultimately has attracted the health-promoting effects of the natural products to be used as medicinal products (Cvetanović et al., 2018). Many plants having culinary uses have been detailed scientifically against worldwide common diseases like diabetes mellitus (DM), Alzheimer's, and cancer, owing to their medicinal properties. Nowadays, it is the need for such drugs that are safer to use in long-term therapy for these diseases without severe side effects (Mocan et al., 2016). Researchers have focused on developing a scientific rationale among the medicinal or dietary effects and chemical constituents of plants having traditional medicinal uses. There is a particular emphasis on such plants exhibiting antioxidant activities, as these plants are reported to have a lower risk of degenerative diseases, including cardiovascular disorders and cancer (Pérez-Jiménez et al., 2008). Researchers are exploiting an easy and cost-effective tactic toward the selection of medicinal plants by studying their traditional folklore uses among different ethnic groups, which is then followed up by their *in-vitro* and *in-vivo* tests or biological validation, leading toward the discovery of novel bioactive molecules. These biologically active molecules are further studied and modified to be used as therapeutic moieties (McRae et al., 2007).

Oxidative stress is caused by the generation of highly reactive oxygen species, which plays a key role in the pathogenesis of many physiological illnesses, including cell injury, cancer, and hepatic, cardiac, neurological, and renal problems (Oladejo et al., 2019). As a result of increased susceptibility of humans to various forms of lethal diseases, there has been a globally emerging trend toward the use of medicinal and nutritional plants as therapeutic antioxidants. In fact, an inverse relationship has been demonstrated between the dietary intake of antioxidant-rich medicinal plants and the prevalence of human diseases (Mahomoodally et al., 2021).

*Sphaeranthus indicus* L. (Asteraceae) is a strongly scented, branched, and annual erect, with branched tapering roots, medicinal herb widely distributed throughout the continents of Australia and Asia (Vikani et al., 2008). This plant has traditional uses against several ailments, for example, dried and powdered leaves of *S. indicus* are useful in the treatment of chronic skin diseases, urethral discharges, and jaundice (Saidapur, 1978). The extract of this plant is diuretic, styptic, and is believed to be beneficial against gastric and liver disorders (Chadha, 1976). Seeds and roots are used as anthelmintic and stomachic (Said and Kenawy, 1956). This plant is also believed to be used for skin disorders as a blood purifier (Kirtikar and Basu, 1918). The extract of this plant is also reported to inhibit the activity of the hyaluronidase enzyme and also possesses antibacterial activity (Lichtenbelt et al., 1998). The phytochemical analysis conducted on this plant has revealed the presence of flavonoids, carbohydrates, mucilage, alkaloids, and gums (Kokate, 1986). Another study has shown that it contains sesquiterpenes possessing anti-inflammatory and immune-stimulating activities (Sadaf et al., 2006). *S. indicus* has also been studied for its antimicrobial and antioxidant activities (Tandon and Gupta, 2020). Previously conducted review articles have highlighted the importance of this plant to have been used in various diseases, including epilepsy, mental illness, hemicrania, jaundice, hepatopathy, diabetes, leprosy, fever, pectoralgia, cough, gastropathy, hernia, hemorrhoids, helminthiasis, dyspepsia, and skin diseases, and have also discussed the important classes of phytochemicals present in this plant (Galani et al., 2010a; Makhija et al., 2011).

Keeping in view the abovementioned traditional uses, this study was conducted to evaluate the reactivity of different solvent extracts/fractions of *S. indicus* flowers. The detailed phytochemical profiling was established *via* determining the total phenolic contents (TPC) and total flavonoid contents (TFC), high-performance liquid chromatography–photo diode array (HPLC-PDA) polyphenolic quantification, and ultra-high-performance liquid chromatography–mass spectrometry (UHPLC-MS) secondary metabolites composition. Similarly, the antioxidant activity of the tested extracts was tested using radical scavenging [2,2-diphenyl-1-picrylhydrazyl (DPPH) and 2,2′-azino-bis-(3-ethylbenzothiazoline-6-sulfonic acid) (ABTS)], reducing power [ferric reducing antioxidant power (FRAP) and cupric reducing antioxidant capacity (CUPRAC)], total antioxidant capacity (phosphomolybdenum assay), and metal chelation activities. Similarly, the key enzyme inhibition potential against the clinically relevant enzymes included in the most common pathologies, i.e., neurological problems [acetylcholinesterase (AChE) and butyrylcholinesterase (BChE)], diabetes (amylase and glucosidase), *Helicobacter pylori*–related infections (urease), and skin disorders (tyrosinase) was also investigated. Furthermore, in order to observe any co-relationships among the tested extracts and biochemical assays activities, exploratory multivariate statistical analyses, i.e., principal component analysis (PCA) and hierarchical clustered analysis (HCA), were also performed. To the best of the literature studied, this study could be the foremost attempt to explore the possible positive effects of this medicinal plant.

## 2 Materials and Methods

### 2.1 Plant Collection, Extraction, and Fractionation

Shade dried flowers of *S. indicus* were obtained from the herbal clinic of the University College of Conventional Medicine (UCCM), Department of Eastern Medicine and Surgery, The Islamia University of Bahawalpur, Bahawalpur (Voucher Number 67/LS-16/X/17). The flowers of *S. indicus* were washed with cold water and placed for air drying, and finally ground into a fine powder using a kitchen mill grinder. The powdered drug was passed through a 50-mesh sieve (297 µm) and soaked in methanol for 24 h. After that, the soaked contents were pressed through a muslin cloth, filtered through Whatman filter paper #42 (pore size 2.5 µm). The solvent was evaporated through a rotary evaporator (Heidolph Co. Ltd., Japan). The dried methanol extract was further dispersed in distilled water and was sequentially fractionated with *n*-hexane, benzene, chloroform, ethyl acetate, and *n*-butanol solvents. All the resultant fractions were dried using the rotary evaporator.

### 2.2 Phytochemical Composition

#### 2.2.1 Total Bioactive Contents

The total bioactive contents of all the extracts/fractions were determined by evaluating the TPC and TFC. The TPC was determined by the Folin–Ciocalteu method as reported previously, and the results were expressed as milligram gallic acid equivalent per gram of extract (mg of GAE/g extract). Similarly, the aluminum chloride colorimetric method, as reported earlier, was utilized to determine the TFC (Wolfe et al., 2003). The results of TFC were reported as milligrams of quercetin equivalent per gram of extract (mg QE/g extract).

#### 2.2.2 HPLC-PDA Polyphenolic Quantification

A list of 22 different polyphenolic standards was tested to be quantified in all the samples using HPLC-PDA analysis as reported previously. The analysis was performed on a Waters liquid chromatograph equipped with a model 600 solvent pump and a 2996 PDA detector, and Empower v.2 Software (Waters Spa, Milford, MA, United States) was used for the acquisition of data (Locatelli et al., 2017).

#### 2.2.3 UHPLC-MS Analysis

UHPLC-MS analysis of methanol and ethyl acetate extracts was performed (negative ionization mode) on the Agilent 1290 Infinity LC system coupled with Agilent 6520 Accurate-Mass Q-TOF mass spectrometer with dual ESI source, as reported earlier (Saleem et al., 2019b). The METLIN database was used for the tentative identification of different secondary metabolites in the tested samples.

### 2.3 Biological Assays

#### 2.3.1 Antioxidant Activity

Antioxidant potential of all the extract/fractions was evaluated by free radical scavenging (DPPH, ABTS), reducing antioxidant power (FRAP, CUPRAC), phosphomolybdenum, and metal chelation assays using the standard procedures, as has been reported previously (Ahmad et al., 2019). All the antioxidant assay results (except metal chelation) were expressed as milligrams of Trolox (water-soluble vitamin E analog) equivalent per gram of dry extract (mg TE/g extract), while ethylenediaminetetraacetic acid equivalent (mg EDTAE/g extract) was used for metal chelation assay. Chemical antioxidant assays, like the DPPH assay, are of no pharmacological relevance because they are chemical tests, and there is no evidence for health therapeutic benefits based on such chemical assays.

#### 2.3.2 Enzyme Inhibition Assays

The inhibition potential of the methanolic extract and all other fractions was tested against α-amylase, α-glucosidase, urease, cholinesterases (AChE and BChE), and tyrosinase enzymes using standard *in-vitro* assays as reported previously (Grochowski et al., 2017; Ahmad et al., 2019). The results of the enzyme inhibition assays were presented as percentage inhibition against all the enzymes except for α-amylase, which was recorded as millimoles (mmol) of acarbose equivalents per gram of extract (ACAE/g). The percentage enzyme inhibition for all the tested enzymes except α-amylase was measured using the following equation:

$$Inhibiton\;\left(\%\right)=\left(\frac{Abs.\;of\;control-Abs.of\;sample}{Abs.\;of\;control}\right)\times 100$$

### 2.4 Statistical Analyses

All the assays were performed in triplicate and presented as average, with standard deviation. Standard curve, correlation coefficient (R^2^), statistical comparison of means using one-way analysis of variance, all regarded *p* < 0.05 as significant. PCA and HCA were achieved to gain insights into the variability between extraction solvents in terms of the evaluated bioactivities. SPSS and R software v. 3.6.2 with FactoMineR was used for this purpose.

## 3 Results and Discussion

### 3.1 Total Bioactive Contents and Phytochemical Composition

Phytochemicals, specifically phenols and flavonoids, are usually considered as most bioactive secondary metabolites in plants, which are also the defensive compounds that are produced as a response to environmental stress (Rahman et al., 2018). In this study, different solvent extract/fractions of *S. indicus* were assessed for their TPC and TFC, and the obtained results are mentioned in Table 1. The highest value of TPC was found for methanolic extract (94.07 mg GAE/g), while the *n*-butanol fraction showed the lowest phenolic contents. Similarly, for flavonoid contents determination, a similar pattern to that of the TPC was noted, and the methanol extract showed maximum TFC value, i.e., 78.70 mg QE/g extract.

**TABLE 1:** Total bioactive contents of *S. indicus* extracts/fractions.

Samples	TPC (mg GAE/g extract)	TFC (mg QE/g extract)
Methanol	94.07 ± 0.28	78.70 ± 0.67
*n*-hexane	55.85 ± 0.25	40.02 ± 0.35
Benzene	55.97 ± 0.07	51.79 ± 0.26
Chloroform	73.36 ± 0.20	57.71 ± 0.18
Ethyl acetate	77.33 ± 0.25	72.36 ± 0.59
*n*-butanol	28.27 ± 0.20	37.22 ± 0.53

Data from the three repetitions are mean ± S.D. GAE: gallic acid equivalent; QE: Quercetin equivalent.

**FIGURE 1:**
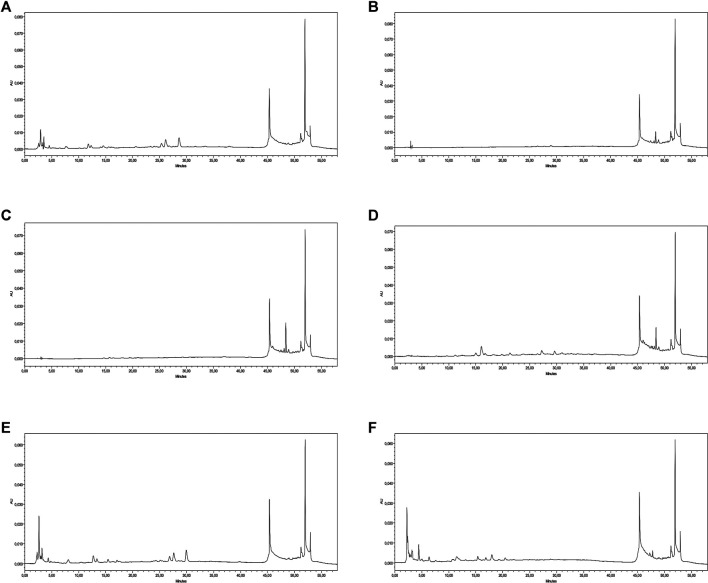
HPLC-PDA chromatograms. **(A)** methanol, **(B)** *n*-hexane, **(C)** benzene, **(D)** chloroform, **(E)** ethyl acetate, and **(F)** *n*-butanol) of polyphenolics quantified in the tested extracts.

Similarly, to gain a more in-depth insight into the phytochemical composition of the studied plant, a list of 22 important standard phenolic phytochemicals were tested for their quantification in all the extracts/fractions of *S. indicus*; however, all the studied extracts were found to be quantified for nine of these compounds. The results of these quantified phenolics are presented in Table 2, and their respective HPLC-PDA chromatograms are shown in Figure 1. From the results, it could be seen that *S. indicus* methanol extract contained a higher amount of phenolics in comparison with the other extracts, with the highest amounts of rutin (6.45 μg/g extract) and naringenin (3.82 μg/g extract), while 3-hydroxybenzoic (0.54 μg/g extract) and sinapinic acid (0.36 μg/g extract) were quantified in lesser amounts, and epicatechin was detected as below the limit of detection (BLD). Likewise, the *n*-butanol extract contained gallic acid (1.61 μg/g extract), epicatechin (1.43 μg/g extract), and 3-OH-4-MeO benzaldehyde (0.21 μg/g extract). Interestingly, none of the tested phenolic standards were present in the *n*-hexane extract, and the benzene extract was found to contain only *t*-ferulic acid (BLD), which may be due to the nonpolar nature of these extracts. The ethyl acetate extract was found to contain two compounds, including chlorogenic acid (2.58 μg/g extract) and gallic acid (0.35 μg/g extract). Similarly, epicatechin (0.99 μg/g extract), 3-hydroxybenzoic acid (1.65 μg/g extract), and naringenin (0.66 μg/g extract) were quantified in the chloroform extract. Overall, this phenolic profiling confirms the presence of important secondary metabolites, so these plant extracts/fractions can be explored further for the isolation of bioactive molecules having potential important activities.

**TABLE 2:** HPLC-PDA polyphenolic quantification of *S. indicus* extracts/fractions.

Samples	Polyphenolics quantified								
	Gallic acid	Chlorogenic acid	Epicatechin	3-hydroxybenzoic acid	3-OH-4-MeO benzaldehyde	Rutin	Sinapinic acid	*t*-ferulic acid	Naringenin
Methanol	nd	nd	BLD	0.54 ± 0.04	nd	6.45 ± 0.68	0.36 ± 0.04	nd	3.82 ± 1.02
*n*-hexane	nd	nd	nd	nd	nd	nd	nd	nd	nd
Benzene	nd	nd	nd	nd	nd	nd	nd	BLD	nd
Chloroform	nd	nd	0.99 ± 0.88	1.65 ± 0.87	nd	nd	nd	nd	0.66 ± 0.07
Ethyl acetate	0.35 ± 0.04	2.58 ± 0.99	nd	nd	nd	nd	nd	nd	nd
*n*-Butanol	1.61 ± 0.11	nd	1.43 ± 0.53	nd	0.21 ± 0.02	nd	nd	nd	nd

All values expressed are means ± S.D. of three parallel measurements; nd: not detected; BLD: below limit of detection.

**FIGURE 2:**
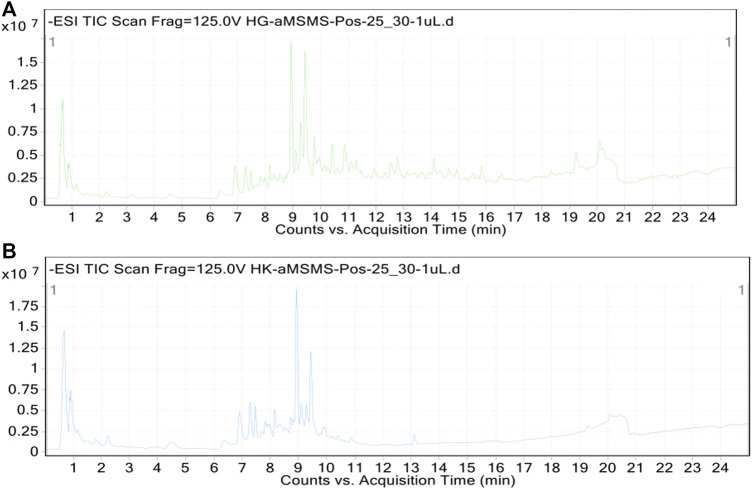
Total ion chromatograms (TICs) of *S. indicus* methanol **(A)** and ethyl acetate **(B)** extracts.

Furthermore, to have detailed individual secondary metabolites profiling, UHPLC-MS analysis was performed on the methanol and ethyl acetate extract/fraction (both of these extracts were found to have higher phenolic and flavonoid contents). Standard total ion chromatograms of the methanol and ethyl acetate extract/fraction with mass spectrometric peaks are shown in Figures 2A,B, respectively. Whereas the list of individual secondary metabolites as tentatively identified in the methanol and ethyl acetate extract/fraction is presented in Tables 3, 4, respectively. As indicated in Table 3, the methanolic extract revealed the tentative presence of 22 different secondary metabolites, and most of these were phenolic and flavonoid derivatives, including quinic acid, benzoic acids, 7,8-dihydroxycoumarin, 3′-(6″-galloylglucosyl)-phloroacetophenone, robinetin 3-rutinoside, agecorynin C, absindiol, and moreollin. Similarly, the ethyl acetate fraction showed the tentative identification of 10 different secondary metabolites, including 10-acetoxyligustroside, 2,4,6-trihydroxybenzoic acid, syringin, and robinetin 3-rutinoside belonging to phenols and flavonoids classes. As far as our literature search, this is the first report on such detailed phytochemical profiling of this plant.

**TABLE 3:** UHPLC-MS analysis of *S. indicus* methanol extract (negative ionization mode).

Serial No.	Possible compound	Compound class	Mol. Formula	Mol. Mass	TR (min)	B. Peak (*m/z*)
1	Quinic acid	Phenol	C_7_H_12_O_6_	192.06	0.665	191.05
2	2,3′,4,6-Tetrahydroxy-benzophenone	Xanthones	C_13_H_10_O_5_	246.05	0.692	245.04
3	2,4,6-Trihydroxybenzoic acid	Phenol	C_7_H_6_O_5_	170.022	1.557	169.01
4	3,4-Dihydroxybenzoic acid	Phenol	C_7_H_6_O_4_	154.027	3.179	153.01
5	Scopolin	Phenolic	C_16_H_18_O_9_	354.1	4.582	353.08
6	Vanilloloside	Glycoside	C_14_H_20_O_8_	316.117	7.228	315.1
7	7,8-Dihydroxycoumarin	Flavonoid	C_9_H_6_O_4_	178.027	7.454	177.01
8	Syringin	Phenol	C_17_H_24_O_9_	372.14	7.819	371.13
9	3'-(6″-Galloylglucosyl)-phloroacetophenone	Flavonoid	C_21_H_22_O_13_	482.11	7.902	481.1
10	4-*p*-Coumaroylquinic acid	Phenol	C_16_H_18_O_8_	338.102	8.005	337.09
11	Thevetin B	Glycoside	C_42_H_66_O_18_	858.43	8.159	857.42
12	Robinetin 3-rutinoside	Flavonoid	C_27_H_30_O_16_	610.15	8.722	609.15
13	Formononetin 7-(6″-methylmalonylglucoside)	Flavonoid	C_26_H_26_O_12_	530.14	9.778	529.14
14	Ferulic acid	Phenol	C_10_H_10_O_4_	194.059	9.833	193.051
15	Agecorynin C	Flavonoid	C_22_H_24_O_9_	432.14	9.96	431.13
16	Tetracaffeoylquinic acid	Phenol	C_43_H_36_O_18_	840.19	10.882	839.18
17	5,8,12-trihydroxy-9-octadecenoic acid	Fatty acid	C_18_H_34_O_5_	330.24	10.977	329.23
18	Coriandrone D	Phenol	C_18_H_24_O_8_	352.15	11.537	351.15
19	9,16-dihydroxy-palmitic acid	Fatty acid	C_16_H_32_O_4_	288.23	11.756	287.22
20	Cinnamodial	Sesquiterpene	C_17_H_24_O_5_	308.163	11.951	307.155
21	Absindiol	Phenol	C_15_H_22_O_4_	266.15	11.955	265.144
22	Moreollin	Flavonoid	C_35_H_42_O_8_	590.29	14.635	589.28

TR: retention time; B. peak: base peak.

**TABLE 4:** UHPLC-MS analysis of *S. indicus* ethyl acetate fraction (negative ionization mode).

Sr. no	Possible compound	Compound class	Mol. Formula	Mol. Mass	TR (min)	B. Peak (*m/z*)
1	Lippioside I	Phenol	C_25_H_30_O_13_	538.16	0.653	537.16
2	Theobromine	Alkaloid	C_7_H_8_N_4_O_2_	180.07	0.67	179.06
3	10-Acetoxyligustroside	Phenol	C_27_H_34_O_14_	582.19	0.872	581.19
4	2,4,6-Trihydroxybenzoic acid	Phenol	C_7_H_6_O_5_	170.022	1.543	169.015
5	Scopolin	Phenolic Glycoside	C_16_H_18_O_9_	354.1	4.498	353.088
6	Vanilloloside	Glycoside	C_14_H_20_O_8_	316.12	7.223	315.11
7	Syringin	Phenol	C_17_H_24_O_9_	372.14	7.816	371.135
8	Thevetin B	Glycoside	C_42_H_66_O_18_	858.43	8.157	857.42
9	Robinetin 3-rutinoside	Flavonoid	C_27_H_30_O_16_	610.15	8.721	609.15
10	Tetracaffeoylquinic acid	Phenol	C_43_H_36_O_18_	840.19	10.876	839.18

TR: retention time; B. peak: base peak.

### 3.2 Antioxidant Activity

Free radicals are the agents that cause gene mutation and the transformation of molecules in the body. These reactions can be controlled by antioxidant defensive mechanisms in living organisms. A number of natural extracts and products are used as food supplements and as topical applications for their antioxidant activity. This requires further investigation of such extracts and products for their source and the compounds responsible for antioxidant activities (Dai and Mumper, 2010).

In the current research, the antioxidant activity of *S. indicus* extract/fractions was determined *via* six different assays (DPPH, ABTS, CUPRAC, FRAP, phosphomolybdenum, and metal chelation activity), and the results are presented in Table 5. Overall, it was noted that the methanolic extract having the highest bioactive contents showed maximum values for radical scavenging and reducing power assays. This can be correlated to the presence of high amounts of phenols and flavonoids in this extract, as a positive relationship between bioactive components with reducing power and free radical scavenging as reported previously (Khan et al., 2019). While, for phosphomolybdenum assay, the benzene fraction showed the highest value, i.e., 5.33 mg TE/g, and the *n*-hexane fraction exerted the highest value for metal chelation activity (12.64 mg EDTA/g). Our results are in line with previous studies highlighting the considerable antioxidant activity of this plant (Shirwaikar et al., 2006; Tiwari and Khosa, 2009; Galani et al., 2010a; Kavitha and Satish, 2015; Vijayalakshmi and Rao, 2019). A flavonoid 5-hydroxy-7-methoxy-6-C-glycosylflavone with remarkable antioxidant potential has also been previously isolated from this plant (Mishra et al., 2007). Likewise, Tiwari and Khosa, (2009) reported the *in-vivo* antioxidant potential of methanolic extract of *S. indicus*, by increasing the levels of superoxide dismutase, catalase, and glutathione peroxides involved in the mechanism of reducing malondialdehyde levels in rats (Tiwari and Khosa, 2009). Similarly, many sesquiterpenes (including 2-hydroxycostic acid, eudesmenolide, and sphaeranthanolide) having antioxidant potential have also been reported in *S. indicus* (Galani et al., 2010b). HPLC polyphenolic quantification and UHPLC-MS analysis in the current study also show the presence of important phytocompounds like rutin and naringenin; both of these flavonoids have reported antioxidant potential (Yang et al., 2008; Cavia-Saiz et al., 2010). Likewise, some of the other compounds identified by phytochemical profiling, including quinic acid, ferulic acid, syringin, and coumarin derivative, have also been reported to have strong antioxidant properties (Hung et al., 2006; Srinivasan et al., 2007; Kostova et al., 2011; Ahmad et al., 2015). These compounds might be responsible for the antioxidant and reducing activities exhibit by the methanolic extract of *S. indicus*. Benzene and *n*-hexane fractions showed the highest value for phosphomolybdenum and metal chelation assays, respectively. This revealed no linear correlation with the bioactive constituents of the said fractions. Literature supports the theory that this trend might be possible due to the presence of some non-phenolic compounds in these fractions (Saleem et al., 2019a; Khurshid et al., 2019; Zengin et al., 2019).

**TABLE 5:** Antioxidant activities of *S. indicus* extracts/fractions.

Samples	DPPH (mg TE/g)	ABTS (mg TE/g)	CUPRAC mg TE/g	FRAP (mg TE/g)	Phosphomolybdenum (mg TE/g extract)	Metal chelating (mg EDTAE/g extract)
Methanol	235.66 ± 2.71	206.81 ± 4.41	458.2 ± 6.31	241.28 ± 1.93	2.66 ± 0.23	4.63 ± 1.35
*n*-Hexane	14.74 ± 3.62	Na	120.68 ± 6.77	55.79 ± 0.04	4.74 ± 0.39	12.64 ± 0.89
Benzene	30.94 ± 0.85	Na	115.33 ± 1.63	71.98 ± 0.10	5.33 ± 0.17	7.65 ± 0.41
Chloroform	132.62 ± 2.39	116.61 ± 3.44	253.72 ± 2.46	125.89 ± 1.64	4.94 ± 0.22	9.20 ± 0.86
Ethyl acetate	110.73 ± 3.65	69.90 ± 4.58	187.56 ± 0.16	129.55 ± 5.29	0.81 ± 0.05	3.59 ± 0.56
*n*-Butanol	123.49 ± 2.39	130.83 ± 4.54	185.25 ± 2.57	137.27 ± 2.32	1.05 ± 0.12	4.52 ± 0.15

a TE: trolox equivalent; EDTAE: EDTA equivalent. All values expressed are means ± S.D. of three parallel measurements; na: not active.

### 3.3 Enzymatic Assays

An increasing trend toward the utilization of natural products for treating some common diseases like diabetes, gastritis, gastroduodenal ulcers, Alzheimer's disease, and hyperpigmentation has been observed (Zengin et al., 2016; Khurshid et al., 2019). In this study, the methanolic extract and various fractions of *S. indicus* were evaluated against α-amylase, α-glucosidase, urease, AChE, BChE, and tyrosinase enzymes. The results of the enzyme inhibition assays are presented in Table 6.

**TABLE 6:** Enzyme inhibition studies of *S. indicus* extracts/fractions.

Samples	AChE	BChE	α-glucosidase	α-amylase	Urease	Tyrosinase
Methanol	82.78 ± 0.57	47.68 ± 0.58	87.16 ± 0.17	0.67 ± 0.01	75.63 ± 0.74	83.18 ± 2.16
*n*-Hexane	71.46 ± 0.72	83.74 ± 0.57	12.62 ± 0.12	0.80 ± 0.03	13.46 ± 0.45	51.37 ± 1.05
Benzene	68.53 ± 0.74	78.46 ± 0.85	23.57 ± 0.53	0.71 ± 0.03	29.87 ± 0.53	63.41 ± 3.17
Chloroform	81.72 ± 0.65	81.65 ± 0.76	37.85 ± 0.21	0.76 ± 0.01	61.42 ± 0.68	79.86 ± 1.23
Ethyl acetate	42.56 ± 0.57	82.57 ± 0.68	42.79 ± 0.27	0.48 ± 0.01	82.35 ± 0.79	86.13 ± 2.81
*n*-Butanol	39.24 ± 0.26	34.23 ± 0.62	17.53 ± 0.43	0.44 ± 0.02	37.71 ± 0.46	31.48 ± 1.01
Acarbose	nt	nt	95.83 ± 0.16	nt	nt	nt
Eserine	91.27 ± 1.17	82.82 ± 1.09	nt	nt	nt	nt
Thiourea	nt	nt	nt	nt	98.21 ± 0.18	nt
Arbutin	nt	Nt	nt	nt	nt	84.6 ± 1.9
Kojic acid	nt	Nt	nt	nt	nt	87.24 ± 2.43

Concentration of standards were set at 1 mM; Values expressed are means ± S.D. of the three parallel measurements; nt: not tested. Eserine was used as the standard for AChE and BChE, acarbose for α-glucosidase and α-amylase; thiourea for urease; arbutin and kojic acid for tyrosinase enzymes.

DM is considered to be a global epidemic. It is considered to be at the 8th position for deaths worldwide. An estimated 425 million adults had diabetes worldwide in 2017, and this number is predicted to rise to 629 million by 2045 (Gomes et al., 2019). Oligosaccharides and disaccharides in the small intestine are converted into glucose by the α-glucosidase enzyme. Inhibition of this enzyme reduces the rate of carbohydrate digestion and due to which its absorption is also delayed in the digestive tract. Inhibitors of α-glucosidase have the potential to prevent the development of type-2 DM (Liu et al., 2011). As presented in Table 6, a considerable α-glucosidase inhibition was shown by the methanolic extract (87.16%), and the *n*-hexane fraction was most active against α-amylase (0.80 ± 0.03 mmol ACAE/g extract), whilst all the other fractions showed the least inhibition. The higher α-glucosidase inhibition by the methanol extract can be due to the higher number of flavonoid compounds in this extract, as previously, this class of phytochemicals have been reported for α-glucosidase potential (Liu et al., 2014).

Urease is an enzyme that hydrolysis urea and produces ammonia and carbon dioxide. Gastritis and gastroduodenal ulcer can be caused by *H. pylori*, and urease enzymes aid the growth of these bacteria in the acidic environment of the stomach. Urease produces a cloud of ammonia that is used by the bacterium for its protection (Liaqat et al., 2017); thus the inhibition of this enzyme is important to control *H. pylori*–related infections. In this study, the maximum urease inhibition was shown by the ethyl acetate fraction (82.35%). This phenomenon might be due to the presence of chlorogenic acid in the ethyl acetate fraction as quantified by the HPLC-PDA analysis, which has been previously reported for urease inhibition activity (Xiao et al., 2012).

Similarly, neurodegenerative disorders are a significant health alarm in several developed countries where the elderly population face abnormal emotional changes (Rajakumar et al., 2017). AChE inhibitors are used for the symptomatic treatment of Alzheimer's disease, for myasthenia gravis, and also in other dementias (Pohanka, 2014). On the other hand, BChE is synthesized in the liver and is reported to have more activity in the human blood than that of AChE (Eddleston et al., 2008). Efforts are in progress to use BChE inhibitors for prophylactic treatment of nerve agent poisoning (Chilukuri et al., 2005). In this study, the methanolic extract showed maximum inhibition for AChE (82.78%), while the *n*-hexane fraction presented the highest BChE (83.74%) inhibition. Likewise, ethyl acetate fraction also showed considerable inhibition of BChE (82.57%).

Tyrosinase enzyme is catalyzed in three steps during melanin biosynthesis. So, tyrosinase inhibitory effects have significance in the cosmetic industry to alter the tone of the skin (Shimizu et al., 2000). Overproduction and accumulation of melanin pigment in the skin can lead to several disorders, including neurodegenerative disorders (Khurshid et al., 2019). The ethyl acetate fraction showed maximum tyrosinase inhibition, i.e., 86.13%, while the methanolic extract showed 83.18% inhibition. The methanolic extract was found to be rich in several phenolic and flavonoid compounds that might be responsible for inhibiting mushroom tyrosinase enzyme, as previously reported studies have indicated the anti-tyrosinase capacity of different phenolics and flavonoids (Bouzaiene et al., 2016; De Freitas et al., 2016; Cespedes et al., 2017; Choi et al., 2019).

### 3.4 Exploratory Multivariate Analyses

**FIGURE 3:**
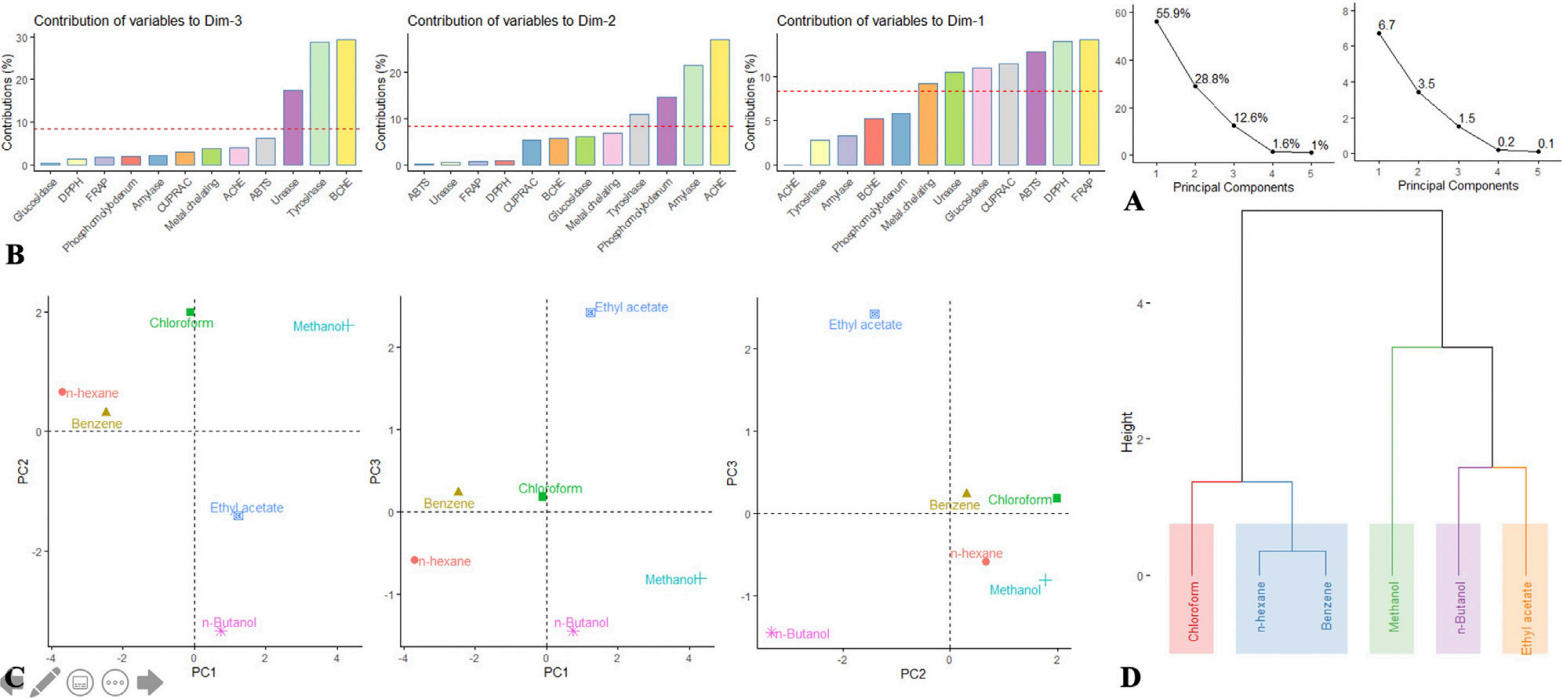
**(A–C)** Principal component analysis. **(A)** Eigenvalue and percentage of explained variance by each dimension. **(B)** Contribution of bioactivities to principal components. **(C)** Distribution of the samples on the factorial plane PC1 vs PC2, PC1 vs PC3, and PC2 vs PC3. **(D)** Dendrogram of HCA.

As has been seen *via* the univariate analysis, there is a high difference among the extraction solvents. To better describe and for greater understanding of the overall activity variations between these solvents, we undertook to perform an exploratory multivariate analysis, i.e., PCA and HCA. Firstly, the data matrix was submitted to PCA with the intention of reducing the dimensionality of the data set by compressing it onto a smaller set of the principal component (PC). The obtained results are given in Figures 3A–C; based on Kaiser Criterion, only the first three components that contained most of the information were selected. PC1 resumed 55.9% of the total information and was largely determined by FRAP, DPPH, ABTS, CUPRAC, and to a limited extent glucosidase (Figure 3B). PC2 captured 28.8% of the information and was linked to AChE, amylase, and to a lesser extent to phosphomolybdenum. PC3 synthetized 12.6% of the information and was bound to BChE, tyrosinase, and urease. In essence, PC1, PC2, and PC3 combined explained almost 97% of the variation in the data set, and the resulting scatter plots PC1 *vs* PC2, PC1 *vs* PC3, and PC2 *vs* PC3 are depicted in Figure 3C. By observing the three scatter plots, a great variability among the extraction solvents can be noted, albeit benzene and *n*-hexane seemed to be close enough. To better reflect the potential clusters, a complementary analysis, i.e., HCA was done. Results depicted in the figure revealed five distinct clusters (Figure 3D).

Both exploratory multivariate analyses allowed to highlight the differential impact of extraction solvents on the biological activities of *S. indicus.* Thus, depending on the solvent employed for recovering the bioactive molecules, extracts obtained from the same material of a plant may differ with respect to their biological activities. Moreover, considering the difference in chemical structure and physicochemical properties of the bioactive molecules, it is impossible to propose a universal standard solvent. Therefore, the selection of the extraction solvent must take into account, the type of molecules expected to be extracted from the plant material, as well as the activities being assessed. In addition, it must be able to preserve the quality of the chemical structure of the desired molecules, as reported previously (Harborne, 1998).

## 4 Conclusion

This research work has highlighted the positive effects by determining the detailed phytochemical and biological composition of different polarity solvent/fractions obtained from *S. indicus* flowers. Phytochemical profiling as achieved by HPLC-PDA and UHPLC-MS analyses has revealed the identification of important secondary metabolites belonging to phenolic, flavonoid, and glycoside classes. The most polar solvent extract/fractions were found to contain the higher bioactive contents. All the tested extracts/fractions showed varying antioxidant and enzyme inhibition potential. Moreover, statistical analyses confirm the relationship among contents and the observed biological activities. Overall, the results obtained by this comprehensive report provide a framework for the utilization of *S. indicus* flower extract/fractions as a natural source for bioactive compounds. However, further work regarding isolation and characterization studies are recommended.

## Data Availability

The original contributions presented in the study are included in the article/supplementary material, further inquiries can be directed to the corresponding author/s.

## Abbreviations

ABTS, 2,2′-azino-bis (3-ethylbenzothiazoline-6-sulfonic acid); AChE, acetylcholinesterase; BChE, butyrylcholinesterase; CUPRAC, cupric reducing antioxidant capacity; DPPH, 2,2-diphenyl-1-picrylhydrazyl; EDTA, Ethylenediaminetetraacetic acid; FRAP, ferric reducing antioxidant power; MTT, 3-4,5-dimethylthiazol-2-yl-2,5-diphenyltetrazolium bromide; PCA, principal component analysis; UHPLC-MS, ultra-high-performance liquid chromatography–mass spectrometry
